# Supplementary material for: Multiscale distribution of oxygen puddles in 1/8 doped YBa2Cu3O6.67
Source: Sci Rep. 2013 Aug 8;3:2383. doi: 10.1038/srep02383 (PMC3737503; doi:10.1038/srep02383)
Supplement: Supplementary Information — Multiscale distribution of oxygen puddles in 1/8 doped YBa2Cu3O6.67 [file srep02383-s1.doc]

SUPPLEMENTARY INFORMATION

**Multiscale distribution of oxygen puddles in 1/8 doped YBa_2_Cu_3_O_6.67_**

Alessandro Ricci^1,4^, Nicola Poccia^2,4^, Gaetano Campi^3^, Francesco Coneri^2^, Alessandra Stella Caporale^3,4^, Davide Innocenti*^4^*, Manfred Burghammer^5^, Martin v. Zimmermann^1^, Gabriel Aeppli^6^, Antonio Bianconi^3,4,*^

*^1^Deutsches Elektronen-Synchrotron DESY, Notkestraße 85, D-22607 Hamburg, Germany.*

*^2^MESA+ Institute for Nanotechnology, University of Twente, P. O. Box 217, 7500AE Enschede, Netherlands*

*^3^Institute of Crystallography, CNR, via Salaria Km 29.300, Monterotondo Stazione, Roma, I-00016, Italy*

*^4^Rome International Center for Materials Science Superstripes RICMASS, via dei Sabelli 119A, 00185 Roma, Italy*

*^5^European Synchrotron Radiation Facility, B. P. 220, F-38043 Grenoble Cedex, France*

*^6^London Centre for Nanotechnology and Department of Physics and Astronomy, University College London, 17–19 Gordon Street, London WC1H 0AH, UK*

*Correspondence to: [antonio.bianconi@ricmass.eu](mailto: <script type%3D'text/javascript'> <!-- var prefix %3D 'ma' + 'il' + 'to'; var path %3D 'hr' + 'ef' + '%3D'; var addy38291 %3D 'antonio.bianconi' + '@'; addy38291 %3D addy38291 + 'ricmass' + '.' + 'eu'; document.write('<a ' + path + '\'' + prefix + ':' + addy38291 + '\'>'); document.write(addy38291); document.write('<\/a>'); //-->\n </script><script type%3D'text/javascript'> <!-- document.write('<span style%3D\'display: none;\'>'); //--> </script>This email address is being protected from spambots. You need JavaScript enabled to view it. <script type%3D'text/javascript'> <!-- document.write('</'); document.write('span>'); //--> </script>)

**Magnetic characterization**

The magnetic behavior vs. temperature of our YBCO sample at 1/8 doping (see Fig. S1) has been characterized by means of the Vibrating Sample Magnetometer (VSM) option in a Physical Properties Measurement System (PPMS 6000) from Quantum Design. Here a linear motor vibrates the sample with a frequency of 40 Hz and amplitude of 2 mm at the center of a pick-up coil, and the induced voltage is measured synchronically with the oscillation. A magnetization measurement has been chosen to consist of the averaged value over 40 data points that is over a period of one second of oscillation. These parameters guarantee a good signal to noise ratio.


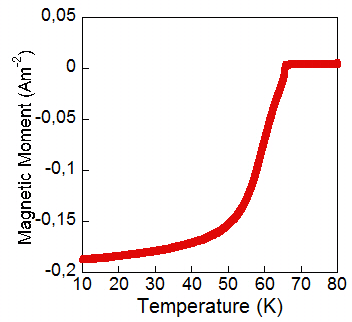


**Figure S1:** The diamagnetic response across the superconductive transition of the YBa_2_Cu_3_O_6.67_ single crystal. After cooling down the sample from room temperature to 10 K in zero applied field (Zero Field Cooling, ZFC) the diamagnetic response upon application of H = 20 Oe has been observed during the warm up across the transition, spanning the 10K to 80K temperature range at a rate of 0.5K/min K. The superconducting transition temperature results to be T_c_ = 66 K.

**Scanning micro X-ray diffraction (µXRD) experimental set up**

The ID13 beam line of the European Synchrotron Radiation Facility (ESRF) is specialized in the delivery of micro-focused x-ray beams for x-ray diffraction experiments. The photon source, in the range 12-13 KeV, is a 18 mm period in vacuum undulator on the ESRF 6.03 GeV storage ring operated in multi-bunch mode with a current of 200 mA. The optics of the micro-focus beamline include compound refractive lenses, Kirkpatrick Baez (KB) mirrors, crossed Fresnel zone plates or waveguides. The ellipsoidal mirror is the main focusing element, demagnifying the source by a factor of 10 (about 40 microns in diameter). The focused beam is defined by a pinhole of 5 micron diameter. The beam is focused by a tapered glass capillary to 1 micron in diameter. The beam-line uses two monochromators positioned in series; the first is a liquid N2 cooled Si-111 double crystal or Si-111 (bounce); the second, is a channel cut monochromator employing a single liquid nitrogen cooled Si crystal. The detector of x-ray diffraction images is a high resolution CCD camera (Mar CCD) with point spread function 0.1 mm, 130 mm entrance window, 16 bit readout placed at a distance of about 90 mm from the sample.

**Scanning nano X-ray diffraction (nXRD) experimental set up**

Scanning nanoscale diffraction experiments were performed at the ID13 beamline of the European Synchrotron Radiation Facility (ESRF), Grenoble, France. The ID13 nano-branch is specialized in the delivery of nano-focused X-ray beams for diffraction experiments. The photon source, an 18 mm period in-vacuum undulator at ESRF works in the range 12-14 KeV with the storage ring operating at 6.03 GeV in the uniform mode with a current of 200 mA. The beamline uses a Si-111 channel cut crystal monochromator cooled with liquid nitrogen. A monochromatic X-ray beam of photon energy 14 KeV (ΔE/E = 10^-4^) was used, which was focused by Kirkpatrick Baez (KB) mirrors to a 300 nm spot size on the sample (full width at half maximum). A 16 bit two-dimensional Fast Readout Low Noise charged coupled device (FReLoN CCD) detector with 2048 x 2048 pixels of 51 x 51 m^2^ was used binned to 512 x 512 pixels. The detector was placed 60 mm behind the sample and offset. Diffraction images were obtained after correcting the 2D images for dark noise, flat field, distortion and eventually background. The CCD camera records the intensity of the satellite superstructures. The interaction volume with the crystal of the 300 nm beam is 1.5 micron.

**High statistical moments analysis**

The higher moments describe particular aspects of a distribution such as how the distribution is asymmetric (Skewness) respect its mean, or peaked (excess of Kurtosis). The third and the forth moment are extremely important to understand how much a distribution deviate from a Normal distribution function that has Skewness (III moment) and Kurtosis (IV moment) both equal to zero.

***Skewness***

The third central moment is a measure of the lopsidedness of the distribution. All the symmetric distributions have a third central moment of zero. The normalized third central moment is called the Skewness generally indicated with γ. A distribution with a negative Skewness parameter is characterized by a heavier tail on the left. A distribution with an heavier tail on the right shows a positive Skewness.

The Skewness of a random variable X is the third standardized moment, denoted γ and defined as

$$\gamma=E\left[\left(\frac{X-\mu}{\sigma}\right)^{3}\right]=\frac{\mu_{3}}{\sigma^{3}}=\frac{E\left[\left(X-\mu\right)^{3}\right]}{\left(E\left[\left(X-\mu\right)^{2}\right]\right)^{3/2}}$$

where μ_3_ is the third moment about the mean μ, σ is the standard deviation, and E is the expectation operator.

The Skewness can be explicated by expanding the previous formula,

$$\begin{matrix} \gamma=E\left[\left(\frac{X-\mu}{\sigma}\right)^{3}\right]=\frac{E\left[X^{3}\right]-3\mu E\left[X^{2}\right]+3\mu^{2}E\left[X\right]-\mu^{3}}{\sigma^{3}} \\ =\frac{E\left[X^{3}\right]-3\mu\left(E\left[X^{2}\right]-\mu E\left[X\right]\right)-\mu^{3}}{\sigma^{3}} \\ =\frac{E\left[X^{3}\right]-3\mu\sigma^{2}-\mu^{3}}{\sigma^{3}} \end{matrix}$$

***Kurtosis***

The fourth central moment is a measure of whether the distribution is peaked and sharp or short and broader, compared to the normal distribution of the same variance. The fourth central moment, where defined, is always non-negative because it is the expectation of a fourth power.

The excess of Kurtosis (κ) is defined to be the normalized fourth central moment minus 3. For a normal distribution κ is always 0. A peaked distribution at the mean with long tails is characterized by high and positive Kurtosis values (leptokurtic). On the contrary bounded distributions tend to have low Kurtosis (platykurtic).

Kurtosis is more commonly defined as the fourth moment around the mean divided by the square of the variance of the probability distribution minus 3:

$$\kappa=\frac{\mu_{4}}{\sigma^{4}}-3$$

That is known as excess of kurtosis respect to the fourth central moment of a normal distribution that is equal to zero.

Suppose that Y is the sum of n identically distributed independent random variables all with the same distribution as X. Then the Kurtosis parameter has the following expression:

$$\kappa\left[Y\right]=\frac{1}{n}\kappa\left[X\right]$$

**The spatial correlation function**

The spatial correlation function G(r) describes the probability of finding an Ortho-VIII domain at a particular distance r from another domain. It follows in general the scaling form $G(r)\approx\frac{1}{r^{\eta}}\exp(\frac{-r}{\xi})$ where ξ is the correlation length, i.e. the typical distance over which an i-O ordering persists, and η is a critical exponent depending on the universality class of the underlying dynamical model associated with the quenching process. To obtain$G(r)$, we have first calculated the correlations of any pairs of spots in the 2D image separated by the vector in the x, y plane, following the expression:

$G(\overrightarrow{r})=\frac{1}{N(\overrightarrow{r})}\sum_{i,j} \delta_{\overrightarrow{r},\overrightarrow{R(i)}-\overrightarrow{R(j)}}\frac{(I_{i}-\left\langle I\right\rangle_{1})(I_{j}-\left\langle I\right\rangle_{2})}{\sigma_{1}\sigma_{2}}$ (1)

where $N(\overrightarrow{r})=\sum_{i,j} \delta_{\overrightarrow{r},\overrightarrow{R(i)}-\overrightarrow{R(j)}}$ indicates the number of points at distance .

The average intensities are calculated according to the expressions

$\left\langle I\right\rangle_{1}=\frac{1}{N(\overrightarrow{r})}\sum_{i,j} \delta_{\overrightarrow{r},\overrightarrow{R(i)}-\overrightarrow{R(j)}}I_{i}$ = $\left\langle I\right\rangle_{2}=\frac{1}{N(\overrightarrow{r})}\sum_{i,j} \delta_{\overrightarrow{r},\overrightarrow{R(i)}-\overrightarrow{R(j)}}I_{j}$ (2)

and the standard deviation are calculated according to:

. (3)

Finally we take $G(r)=\left\langle G(\vec{r}\right\rangle\langle\rangle$ where the average is over all the allowed directions in the grid describing the 2D image.
